# 1,3,4-Thiadiazoles Effectively Inhibit Proliferation of *Toxoplasma gondii*

**DOI:** 10.3390/cells10051053

**Published:** 2021-04-29

**Authors:** Lidia Węglińska, Adrian Bekier, Katarzyna Dzitko, Barbara Pacholczyk-Sienicka, Łukasz Albrecht, Tomasz Plech, Piotr Paneth, Agata Paneth

**Affiliations:** 1 Department of Organic Chemistry, Faculty of Pharmacy, Medical University of Lublin, Chodźki 4a, 20-093 Lublin, Poland; lidia.weglinska@umlub.pl; 2 Department of Molecular Microbiology, Faculty of Biology and Environmental Protection, University of Lodz, Banacha 12/16, 90-237 Lodz, Poland; adrian.bekier@biol.uni.lodz.pl; 3 Institute of Organic Chemistry, Faculty of Chemistry, Lodz University of Technology, Zeromskiego 116, 90-924 Lodz, Poland; barbara.pacholczyk@p.lodz.pl (B.P.-S.); lukasz.albrecht@p.lodz.pl (Ł.A.); 4 Department of Pharmacology, Faculty of Health Sciences, Medical University of Lublin, Chodzki 4a, 20-093 Lublin, Poland; tomasz.plech@umlub.pl; 5 Institute of Applied Radiation Chemistry, Lodz University of Technology, Zeromskiego 116, 90-924 Lodz, Poland; piotr.paneth@p.lodz.pl; 6 International Centre for Research on Innovative Biobased Materials (ICRI-BioM)—International Research Agenda, Lodz University of Technology, Zeromskiego 116, 90-924 Lodz, Poland

**Keywords:** 1,3,4-thiadiazole, cytotoxicity, genotoxicity, *Toxoplasma gondii*, SAR analysis

## Abstract

Congenital and acquired toxoplasmosis caused by the food- and water-born parasite *Toxoplasma gondii* (*T. gondii*) is one of the most prevalent zoonotic infection of global importance. *T. gondii* is an obligate intracellular parasite with limited capacity for extracellular survival, thus a successful, efficient and robust host cell invasion process is crucial for its survival, proliferation and transmission. In this study, we screened a series of novel 1,3,4-thiadiazole-2-halophenylamines functionalized at the C5 position with the imidazole ring (**1b**–**12b**) for their effects on *T. gondii* host cell invasion and proliferation. To achieve this goal, these compounds were initially subjected to in vitro assays to assess their cytotoxicity on human fibroblasts and then antiparasitic efficacy. Results showed that all of them compare favorably to control drugs sulfadiazine and trimethoprim in terms of *T. gondii* growth inhibition (IC_50_) and selectivity toward the parasite, expressed as selectivity index (SI). Subsequently, the most potent of them with *meta*-fluoro **2b**, meta-chloro **5b**, meta-bromo **8b**, meta-iodo **11b** and para-iodo **12b** substitution were tested for their efficacy in inhibition of tachyzoites invasion and subsequent proliferation by direct action on established intracellular infection. All the compounds significantly inhibited the parasite invasion and intracellular proliferation via direct action on both tachyzoites and parasitophorous vacuoles formation. The most effective was *para*-iodo derivative **12b** that caused reduction in the percentage of infected host cells by 44% and number of tachyzoites per vacuole by 93% compared to non-treated host cells. Collectively, these studies indicate that 1,3,4-thiadiazoles **1b**–**12b**, especially **12b** with IC_50_ of 4.70 µg/mL and SI of 20.89, could be considered as early hit compounds for future design and synthesis of anti-*Toxoplasma* agents that effectively and selectively block the invasion and subsequent proliferation of *T. gondii* into host cells.

## 1. Introduction

Congenital and acquired toxoplasmosis caused by the food- and water-born parasite *T. gondii* is one of the most prevalent zoonotic infection of global importance [1,2]. According to World Health Organization (WHO), in the European Region toxoplasmosis affects more than 1 million people each year, resulting only in 2010 in 45 deaths due to congenital toxoplasmosis and almost 73,000 leading causes of disability-adjusted life years [3,4,5]. *Toxoplasma* infection in immunocompetent patients often remains asymptomatic or with symptoms limited to very mild fever, malaise or lymph node swelling and thus rarely requires drug therapy [6,7]. Nevertheless, if primary infection occurs during pregnancy, it can cause serious problems including abortion, stillbirth or, in the newborns, blindness, chorioretinitis, hydrocephalus or microcephalus, and intracerebral calcifications [8]. Important to note, the newborns with vertical *T. gondii* transmission usually have a normal appearance and thus congenital toxoplasmosis is not diagnosed and treated in the first years of life. They later, however, may develop chorioretinitis or neurologic and developmental deficits [9]. Furthermore, in patients with profound T-cell immunodeficiency toxoplasmosis may manifest as potentially fatal encephalitis, pneumonitis, myocarditis and eye diseases, mostly retinochoroiditis [10]. Additionally, it has been well-documented that ocular toxoplasmosis is not necessarily caused through the congenital route but also may result from postnatally acquired *T. gondii* infection [11,12]. Furthermore, in immunocompetent individuals, highly virulent strains of *T. gondii* can cause severe neurological symptoms of acquired toxoplasmosis including epilepsy, schizophrenia and psychosomatic disorders, but causal relationships have not yet been established [13,14].

Currently, the first choice for treatment of toxoplasmosis includes combinations of two antimicrobial drugs, most often inhibitors of dihydrofolate reductase (pyrimethamine and trimethoprim) and dihydropteroate synthetase (sulfadiazine, sulfamethoxazole and sulfadoxine), which block folic acid synthesis [15]. The second-line therapy includes atovaquone, alone or in combination with sulfadiazine, and epiroprim [16]. Although these drugs can cure *T. gondii* infection effectively, up to 80% patients would relapse without a long term of therapy and about 40% patients may discontinue to accepting treatment due to serious side effects, mostly pyrimethamine-related bone marrow suppression, intolerance or allergic reaction to the sulfonamide component [17,18]. In addition, these treatments in clinical practice are highly effective against the tachyzoite stage of the parasite but do not demonstrate activity against the latent bradyzoite cysts that makes toxoplasma a chronic infection [15]. Another challenge of standard therapeutic regimens is that these drugs do not pass through the blood–brain barrier to treat central nervous system infection with *T. gondii* [19]. Though effective vaccine against *T. gondii* has been considered a promising and economical strategy toward preventing and controlling the spread of toxoplasmosis and numerous vaccination trials have been performed for their immunological effects in animal models, there is currently no licensed vaccine available for humans and its development is still far from industrial production [20,21]. To date, the only vaccine available and not suitable for human use is the veterinary Toxovax^®^ licensed for the prevention of *T. gondii* abortions in sheep. This vaccine is based on an attenuated S48 live strain of *T. gondii* with a short shelf life that creates the possibility of reversion of the attenuated mutant to the pathogenic virulent strain, limits production and shelf life, demands cold chain administration, and finally is potentially hazardous to the person administering it [22]. Since a long-term neglect of toxoplasmosis, the serological prevalence of *T. gondii* increasing in the world [23,24]. For this reason, novel drugs with better efficacy and lower toxicity are needed for anti-Toxoplasma therapies.

**Figure 1:**
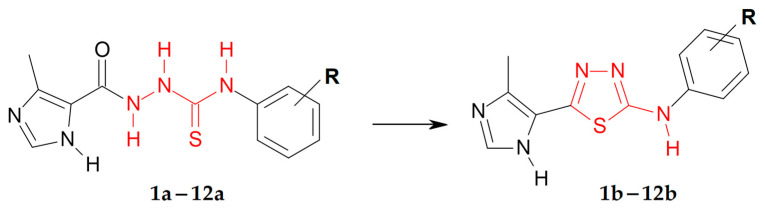
Chemical modifications (red) and structural requirements (black) in this new scaffold.

Recently [25], we published a new series of halophenyl-substituted imidazole thiosemicarbazides with much better and selective anti-*Toxoplasma* effects than sulfadiazine (IC_50s_ 10.30–113.45, selectivity index SI 3.15–19.16, vs. 2721.45 μg/mL, SI < 0.92). Moreover, the most potent of them inhibited tachyzoites proliferation more potently and more selectively than trimethoprim (IC_50_ 12.13 μg/mL, SI > 2.64) and more potently than sulfadiazine in combination with trimethoprim (IC_50_ 37.15 μg/mL). Further results collectively indicated that significant differences in anti-*Toxoplasma* activity correlate with the lipophilicity, with the most lipophilic compounds found to be the most active. Despite promising pharmacological properties of these compounds further enhancing their efficacy and selectivity is important. To achieve this, their linear structure was cyclized to more lipophilic 1,3,4-thiadiazole scaffold (Figure 1), leading to increased antiproliferative activity, presumably due to greater than before cellular uptake, and generally to improving anti-*Toxoplasma* selectivity. Further molecular mechanism investigations from an electron microscopy have shown that these compounds are effective in blocking the entry of the parasite into the host cell and subsequent its division. Thus, the present work provides a description of the activity of these compounds. Initial literature data also indicate that 1,3,4-thiadiazole-type molecules may be promising anti-*Toxoplasma* agents [26]. The limited amount of data, however, indicates the need to explore this approach.

## 2. Materials and Methods

Biological evaluation: All experiments were carried out in BSL-2 laboratory with biosafety cabinet. Compounds **1b**–**12b** were dissolved in dimethyl sulfoxide (DMSO, Sigma-Aldrich, St. Louis, MO, USA) to 75 mM. Sulfadiazine sodium salt (S6387, Sigma-Aldrich) was dissolved in DPBS (Dulbecco’s phosphate buffered saline, D8537, Sigma) without calcium chloride and magnesium chloride. Trimethoprim, pyrimethamine and atovaquone (92131, 46706 and A7986, Sigma-Aldrich), were dissolved in DMSO to 20 mg/mL. The final concentration of DMSO in the compounds and drugs dilutions was not higher than 1.00%. All compounds and drugs were freshly prepared before experiment and sterile filtered. Preliminary in vitro anti-*Toxoplasma* activity data using Vero cells for compounds **3b** and **6b** were reported previously by Góes and coworkers [26].

Cell and parasite culture: The Hs27 (human foreskin fibroblast) (ATCC^®^ CRL-1634™) (ATCC, Manassas, VA, USA) were culturing in DMEM (Dulbecco’s modified Eagle’s medium-ATCC^®^ 30-2002™, ATCC) supplemented with 10% fetal bovine serum (fetal bovine serum, ATCC^®^ 30-2020™, ATCC), 100 I.U./mL penicillin and 100 μg/mL streptomycin (penicillin-streptomycin solution ATCC^®^ 30-2300™, ATCC). Cells were trypsinized (Trypsin-EDTA Solution ATCC^®^ 30-2101™, ATCC) twice a week and seeded at a density of 1 × 10^6^ per T25 cell culture flask and incubated in a 37 °C and 5% CO_2_ to achieve a confluent monolayer. The RH strain of *Toxoplasma gondii* (RH-GFP ATCC^®^ 50940™, ATCC, highly virulent, haplogroup first, expression of green fluorescent protein) was maintained as tachyzoites, in parasite culture medium, which contains DMEM medium with 3% HIFBS (heat-inactivated FBS; 1 h in 56 °C). Infected tissue culture cells were incubated in a 37 °C and 5% CO_2_.

Cytotoxicity assay: Cytotoxicity assay was performed using the tetrazolium salt (MTT, Sigma-Aldrich) and human foreskin fibroblast. For this assay DMEM without phenol red (21063, Gibco, Thermo Fisher Scientific) (Gibco, Thermo Fisher Scientific, Waltham, MA, USA) supplemented with 10% FBS, 100 I.U./mL penicillin and 100 μg/mL streptomycin were used. Briefly, 1 × 10^4^/well of Hs27 cells were placed into 96-well plates and incubated 24 h in 37 °C and 5% CO_2_. Afterwards, an old culture medium was replaced by 100 μL of the compounds or drugs dilutions in culture medium and the cells were treated 24 h. Concentration range of the compounds 0–3000 μM, sulfadiazine 0–25,000 μM, trimethoprim, pyrimethamine and atovaquone 0–250 μM, and DMSO as the solvent 0–20% (data not shown). Then, 50 μL of 1 mg/mL of MTT solution in DMEM without phenol red was added to each well and incubated 2 h (37 °C, 10% CO_2_). Following, cell culture medium was aspirated carefully and 150 μL of DMSO was added to each well, the plates were gently mixed. Then 25 μL 0.1 M glycine buffer (pH 10.5) (Sigma-Aldrich) was added. The optical density at 570 nm on multimode microplate reader SpectraMax^®^ i3 (Molecular Devices, San Jose, CA, USA) was read. The results were expressed as a percentage of viability compared to untreated cells. All experiments were performed in triplicate.

Antiparasitic assay: The influence of compounds **1b**–**12b** and drugs on *T. gondii* RH-GFP proliferation was performed as follows: 1 × 10^4^ per well of Hs27 cells were seeded on black, 96-well, tissue culture-treated plates with optical bottoms (Corning, Wilmington, NC, USA) in DMEM cell culture medium. After 72 h of incubation, the medium was removed and then 1 × 10^5^ per well of tachyzoites of the RH strain were added to the cell monolayers, in DMEM without phenol red with 3% HIFBS. One hour later, the compounds and drugs dilutions in the DMEM without phenol red medium were added to the Hs27 cells with *T. gondii*. Concentration of the compounds below cytotoxic concentration (<CC_30_), sulfadiazine 0–10,000 μM and trimethoprim, pyrimethamine and atovaquone 0–200 μM. After a subsequent 96 h of incubation, the plates were read with covered lids, and both excitation (475 nm) and emission (509 nm) from the bottom using multimode microplate reader SpectraMax^®^ i3. The results were expressed as mean fluorescent intensity (MFI) and transformed to the percentage of viability compared to untreated cells. Finally, the inhibitory concentrations for 50% inhibition of *T. gondii* proliferation (IC_50_) were calculated. All experiments were performed in triplicate.

Influence of Compounds during Toxoplasma Growth (Assay A and B): Hs27 cells were cultured on Lab-Tek™ 4-well Chamber Slides (Nunc, Thermo Fisher Scientific, Waltham, MA, USA) (5 × 10^4^ cells/500 μL per well).

Assay A—after 48 h, the medium was removed and then 5 × 10^5^/500 µL/well tachyzoites of the RH-GFP strain were added to the cell monolayers, in parasite culture medium with the selected compounds at IC_50_ concentration: **2b**, **5b**, **8b**, **11b** or **12b**, for 3 h. Then, the cells were washed to remove extracellular parasites and parasite culture medium (without any compounds) was added for 24 h.

Assay B—after 48 h, the medium was removed and then 5 × 10^5^/500 µL/well tachyzoites of the RH-GFP strain were added to the cell monolayers, in parasite culture medium for 3 h. Then, the cells were washed to remove extracellular parasites, and the cells were treated with the selected compounds at IC_50_ concentration: **2b**, **5b**, **8b**, **11b** or **12b**, for 24 h.

In both assays as the controls, Hs27 cells were infected, but not treated. After subsequent 24 h of incubation (Assay A and B), the slides were washed with sterile PBS, fixed with formaldehyde solution (252549, Sigma-Aldrich), 3.7% in PBS for 20 min and stained with DAPI for 10 min (1 mg/mL, ThermoFisher Scientific). Microscopic analysis of 100 cells in turn, comprised of counting the infected cell ratio and tachyzoites per each parasitophorous vacuoles of infected cell, was performed using a fluorescent microscope (Axio Scope.A1, Carl Zeiss, Jena, Germany) at a magnification ×1000. For image processing ZEISS ZEN Microscope Software version 3.1 was used. Three independent experiments on triplicate chamber slides using the same conditions for both assays were performed.

Kinetics of *T. gondii* growth: The effect of **12b** and drugs on kinetics of *T. gondii* RH-GFP growth was performed as follows: 1 × 10^4^ per well of Hs27 cells were seeded on black, 96-well, tissue culture-treated plates with optical bottoms in DMEM cell culture medium.

Assay 1—After 72 h of incubation, the medium was removed and then 1 × 10^5^ per well of tachyzoites of the RH strain were added to the cell monolayers, in DMEM without phenol red with 3% HIFBS. One hour later, the compound **12b** and drugs dilutions in the DMEM without phenol red medium were added to the Hs27 cells with *T. gondii*.

Assay 2—After 72 h of incubation, the medium was removed and DMEM without phenol red with 3% HIFBS, was added. In the new 96-well, tissue culture-treated plates 2 × 10^5^ per well of tachyzoites of the RH strain were added and treated for 3, and 6 h with the compound **12b** and drugs (diluted in the DMEM without phenol red medium) at 37 °C. Then tachyzoites were wash out three times in DPBS, resuspended in DMEM without phenol red medium with 3% HIFBS, and finally 1 × 10^5^ per well of tachyzoites were added to the Hs27 cells.

The plates were read with covered lids, and both excitation (475 nm) and emission (509 nm) from the bottom using multimode microplate reader SpectraMax^®^ i3. Additionally, fluorescence measurement was performed every 24 h. The plates were incubated at 37 °C with 5% of CO_2_. The results were expressed as mean fluorescent intensity (MFI). The initial value for 1 × 10^5^ of *T. gondii* RH-GFP was 37,000 MFI (log 4.57). The lower limit of detection was 1000 MFI (log 3.0). Data presented as a logarithm of mean fluorescence intensity (log_MFI_) on concentration and time dependent curves. All experiments were performed in triplicate.

Genotoxicity assay: Evaluation of the genotoxic potential of compounds **10b**–**12b** was carried out using a single cell gel electrophoresis technique (comet assay) based on the OxiSelect Comet Assay Kit (Cell Biolabs, Inc., San Diego, CA, USA). All the procedures performed were in line with the manufacturer’s protocol. Human Hs27 cells (fibroblasts), previously incubated with compounds **10b**–**12b**, were mixed with low melting agarose. Cells suspended in agarose were afterwards transferred to glass slides and left for 15 min in cool conditions (at 4 °C). Subsequently, the slides were incubated for 45 min in a lysis buffer (to break down cell membranes) and for the next 30 min in an alkaline solution. Finally, the slides were placed in the electrophoresis chamber, submerged in the TBE buffer and run at room temperature with 1 volt/cm current between the electrodes for 15 min. At the end, the comet agarose gel was dried and the DNA was stained with Vista Green DNA Dye (Cell Biolabs, Inc., San Diego, CA, USA) for 15 min. The images were captured using a fluorescence microscope (Olympus BX63) (Olympus, Tokyo, Japan) combined with XM10 camera (Olympus, Tokyo, Japan). The DNA damages were measured quantitatively using ImageJ software (NIH, Bethesda, MD, USA). At least 50 randomly selected images were used in each analysis.

Graphs and statistical analyses: Statistical analyses and graphs were performed using GraphPad Prism version 9.0.0 for macOS (GraphPad Software, San Diego, CA, USA). For the compounds with the CC_30_ or the IC_50_ values greater than the highest concentration tested, the values were calculated based on extrapolation of the curves. Additionally, a relationship between cytotoxicity and antiparasitic activity were calculated as the ratio of the 30% cytotoxic concentration (CC_30_) to the 50% antiparasitic concentration (IC_50_) and presented as the selectivity index (SI).

## 3. Results and Discussion

### 3.1. Synthetic Chemistry

**Scheme 1:**
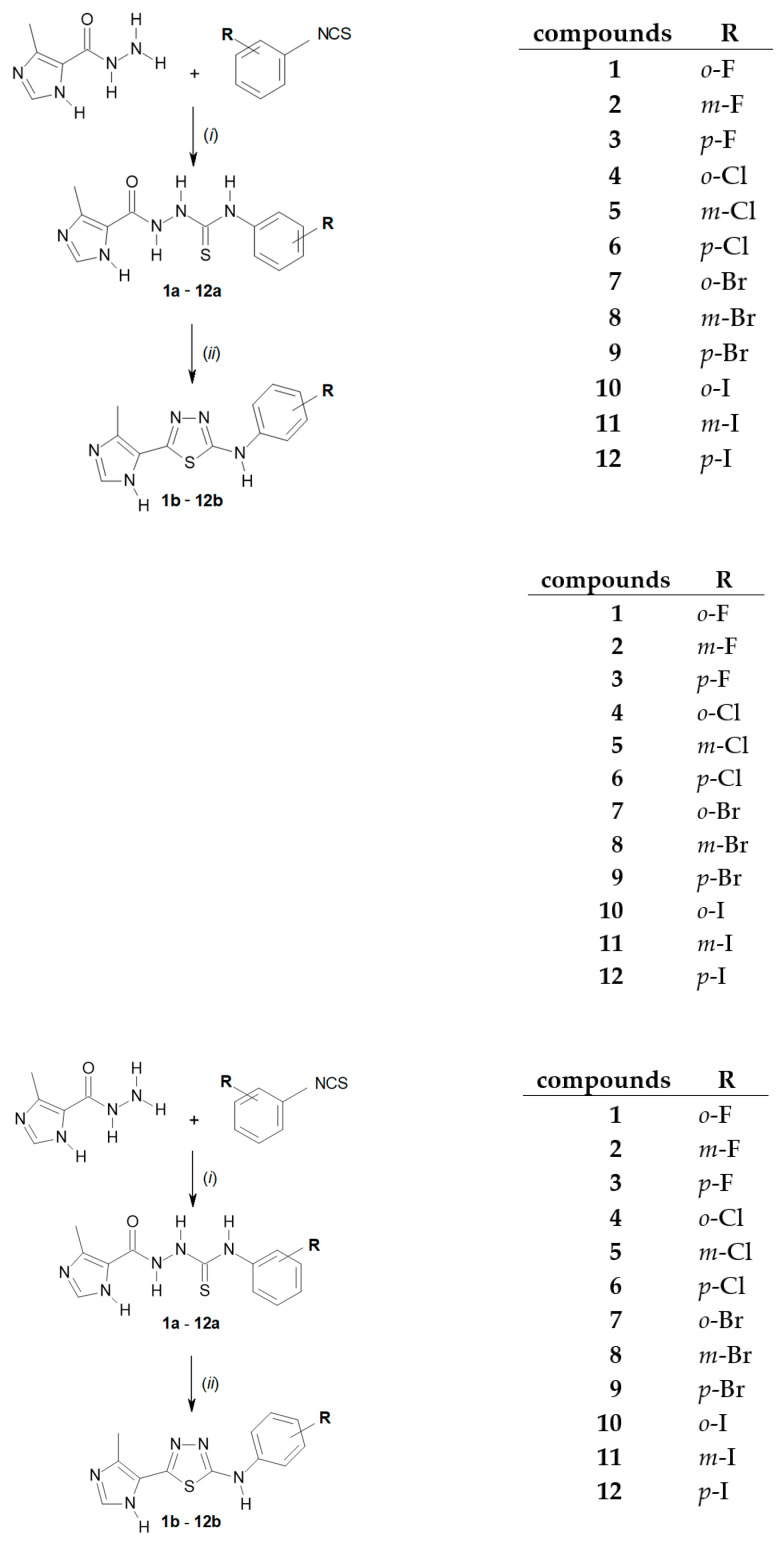
Synthetic route for *N*-halophenyl-(4-methylimidazol-5-yl)-1,3,4-thiadiazole-2-amine (**1b**–**12b**). Conditions: (*i*) EtOH, reflux, 30 min; (*ii*) H_2_SO_4_, rt, 2 h.

The 1,3,4-thiadiazoles (**1b**–**12b**) were prepared under the routine two-step synthetic route (Scheme 1), described previously [25,27]. Shortly, the reaction of the 4-methylimidazole-5-carbohydrazide with related halophenyl isothiocyanate in boiling ethanol gave 1-(4-methylimidazol-5-oyl)-4-halophenylthiosemicarbazides (**1a**–**12a**) in 78–95% yields. Resulting compounds were next dehydrocyclized in the concentrated sulfuric acid to give final products **1b**–**12b** with 18–49% yields. The obtained compounds **1b**–**12b** are stable at room temperature and could be stored for months without decomposition.

All compounds **1b**–**12b** were characterized by ^1^H, ^13^C NMR and IR. The assignment of signals was also confirmed by sets of 2D experiments (^1^H-^1^H COSY,^1^H-^13^C HSQC and ^1^H-^13^C HMBC). Previously, a small series of the products of dehydrative cyclization of 1-(4-methylimidazol-5-yl)-4-arylthiosemicarbazides in the concentrated sulfuric acid has been prepared by Góes et al. [26]. The obtained compounds (**3b**, **6b**) were characterized by ^1^H NMR spectra that revealed the resonance signals at about 10.5 ppm for two NH groups, assigned to the imidazole ring and the amine at C2 position of the 1,3,4-thiadiazole. In the current project, the products **1b**–**12b**, obtained following the same synthetic protocol, were first analyzed in a wet dimethylsulfoxide-d6. As a result, the signals from the NH protons were not observed. However, NMR experiments performed in extra dry DMSO-d6 confirmed that the formation of **1b**–**12b** took place under acidic conditions. Typically, in the ^1^H NMR spectra signals for NH protons are prone to intermolecular exchange processes, which may result in broadening and averaging the signals of multiple NH groups in the sample. Furthermore, the chemical shift of the NH proton may appear at chemical shifts from 4 to 12 ppm depending on the possibility of hydrogen bond formation. The ^1^H NMR spectra of compounds **1b**–**12b** showed signal in the range from 9.98 to 11.91 ppm, corresponding to the NH imidazole ring, while the signal at chemical shift from 4.0 to 7.0 ppm were assigned to the amine at C2 position of the 1,3,4-thiadiazole. Notably, the chemical shift drift for NH proton at C2 position was clearly observed depending on the position of halogen atom on the phenyl ring. In almost all compounds signal from the NH group of imidazole ring was found at about 10.70 ppm, but in case of **4b**, **7b** and **10b** NH tautomerism was observed.

### 3.2. Antiparasitic Activity

**Figure 2:**
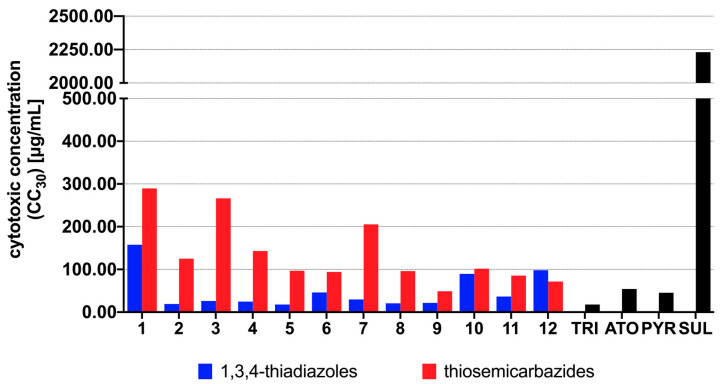
The cytotoxic effects of the thiosemicarbazides **1a**–**12a** and the 1,3,4-thiadiazoles **1b**–**12b** against Hs27 human fibroblast cells. CC_30_ (µg/mL) represents the non-cytotoxic concentration of the compound that caused only 30% of host cells proliferation inhibition in vitro. CC_30_ values were determined based on the plotted curves using GraphPad Prism program (version 9.0.0). Reference drugs: TRI—trimethoprim, ATO—atovaquone, PYR—pyrimethamine, SUL—sulfadiazine.

The 1,3,4-thiadiazoles (**1b**–**12b**) were initially tested to determine their cytotoxic effects against human fibroblast (Hs27) cells line. *T. gondii* is an obligate intracellular parasite, which consequently requires a host cells for growth and proliferation. Therefore, the nontoxic concentrations of the 1,3,4-thiadiazoles **1b**–**12b** considered in this study were those that caused damage only 30% of the Hs27 cells (CC_30_). The CC_30_ results are graphically presented in Figure 2. Details are reported in Supplementary Table S1. The data for previously reported thiosemicarbazides **1a**–**12a**, acyclic precursors of **1b**–**12b**, are also given for comparison.

**Figure 3:**
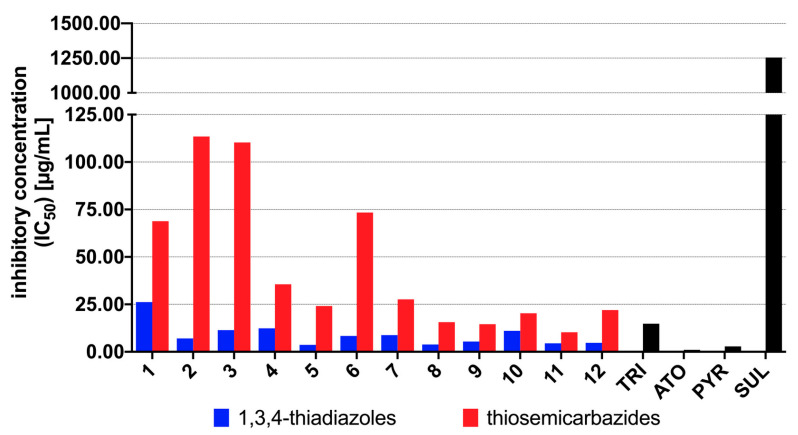
Results of *T. gondii* proliferation assay in vitro for the 1,3,4-thiadiazoles **1b**–**12b** and the thiosemicarbazides **1a**–**12a.** IC_50_ (µg/mL) represents the concentration of the compound required for 50% inhibition of *T. gondii* proliferation in vitro. The IC_50_ values for **1a**–**12a** were taken from Reference [25]. Reference drugs: TRI—trimethoprim, ATO—atovaquone, PYR—pyrimethamine, SUL—sulfadiazine.

**Figure 4:**
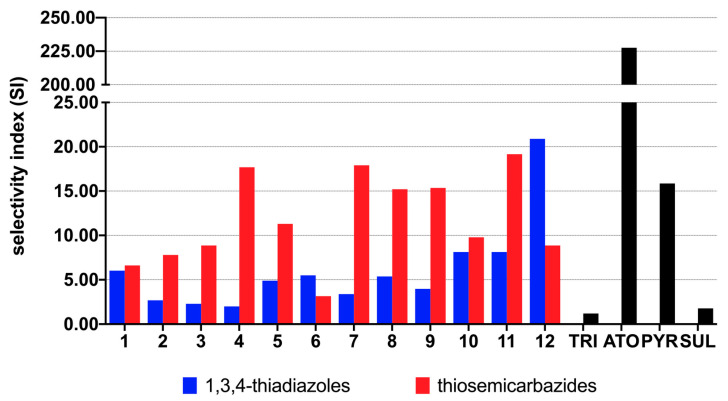
Selectivity index (SI) for the 1,3,4-thiadiazoles **1b**–**12b** and the thiosemicarbazides **1a–12a** calculated as the ratio of CC_30_ to IC_50_. Reference drugs: TRI—trimethoprim, ATO—atovaquone, PYR—pyrimethamine, SUL—sulfadiazine.

Having determined cytotoxic profile of the 1,3,4-thiadiazoles (**1b**–**12b**), we further investigated their efficacy in inhibition of *T. gondii* proliferation. The inhibitory concentration (IC_50_) together with the selectivity index (SI), expressed as a ratio of the cytotoxic concentration (CC_30_) divided by the inhibitory concentration (IC_50_), were determined. Pyrimethamine (PYR) and sulfadiazine (SUL) commonly used for the treatment of human toxoplasmosis [28], atovaquone (ATO) commonly used to treat toxoplasmosis in difficult cases [26] and trimethoprim (TRI) commonly used to treat animals in Europe [29] were used as positive controls. Dimethyl sulfoxide at a concentration of 0.1% was used as the negative control (data not shown). The results for the 1,3,4-thiadiazoles **1b**–**12b** and positive controls are reported in Supplementary Figure S1 and Supplementary Table S1, respectively. Graphical summary of the IC_50_ and SI results is presented in Figure 3 and Figure 4, respectively. The data for **1a**–**12a** are also given for comparison.

As presented in Supplementary Table S1, although higher cytotoxicity of the 1,3,4-thiadiazoles **1b**–**12b** compared to linear precursors **1a**–**12a**, these results indicate that more lipophilic 1,3,4-thiadiazole scaffold (Supplementary Table S2) is promising for anti-*Toxoplasma* activity, as demonstrated by increasing of **1b**–**12b** inhibitory effect on *T. gondii* proliferation. As presented in Figure 3, in all cases more lipophilic 1,3,4-thiadiazoles **1b**–**12b** showed higher potency to inhibit the in vitro growth of *T. gondii* compared with related thiosemicarbazides **1a**–**12a**. Additionally, except for **1b**, all of them proved better anti-*T. gondii* activity and selectivity than SUL and TRI. In all cases the *meta*-isomer showed more activity than its *para* counterpart, which, in turn, was more effective than the isomer *ortho* (*meta* > *para* > *ortho*), indicating relationship between substitution pattern and potency of the 1,3,4-thiadiazoles. No relationship, however, between halogen electron-withdrawing ability and anti-*T. gondii* activity was observed. The best anti-*T. gondii* agents were *meta*-Cl, *meta*-Br and *meta*-I, thereby confirming than substitution pattern rather than electronic or steric effect of halogen controls anti-*T. gondii* response. Although some of the 1,3,4-thiadiazoles showed lower selectivity index than corresponding linear precursors (Figure 4), all of them still exhibited multiple-fold specificities (SI ≥ 2) toward the parasite vs. the host cells. For a drug to be considered a good candidate against toxoplasmosis, its SI should be higher than 1 [30]. Taken together, the findings indicate not only specific anti-*T. gondii* action by the 1,3,4-thiadiazoles but also that their anti-*Toxoplasma* effect was not a result of toxicity to the host cells. Given these results, the 1,3,4-thiadiazoles **1b**–**12b**, especially iodo derivatives **10b** (IC_50_ 11.02 µg/mL, SI 8.13), **11b** (IC_50_ 4.49 µg/mL, SI 8.13) and **12b** (IC_50_ 4.70 µg/mL, SI 20.89) could be considered as early hit compounds for toxoplasmosis therapy.

### 3.3. Genotoxicity

**Figure 5:**
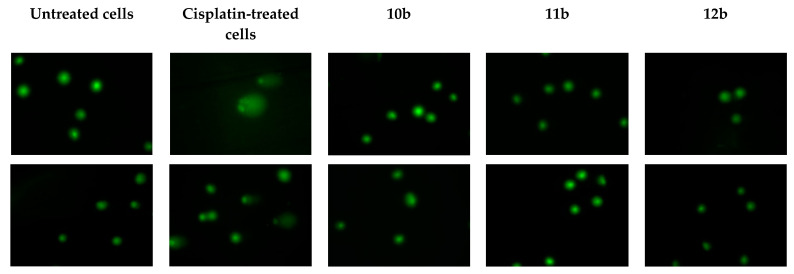
Genotoxicity evaluation of the thiadiazoles **10a**–**12b** in Hs27 cells. The investigated compounds were tested at their CC_30_ concentrations. Cisplatin (positive control) was tested at 10 µg/mL.

**Figure 6:**
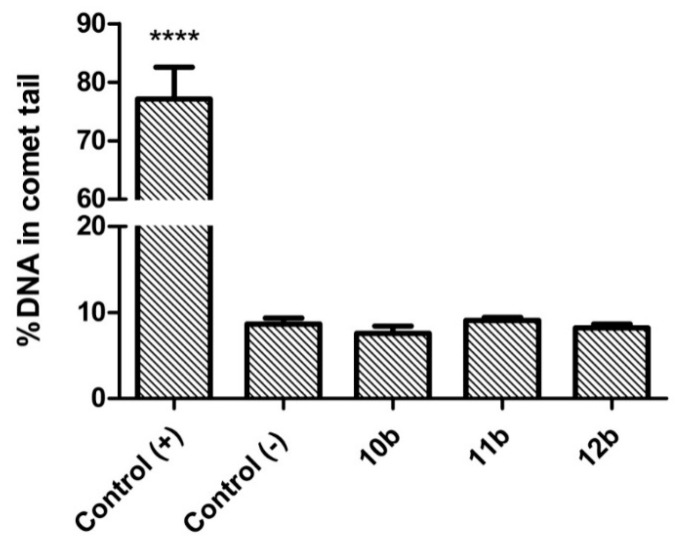
Quantitative analysis of the DNA damage in Hs27 cells after 24 h exposition to cisplatin (positive control, 10 µg/mL) or the thiadiazoles **10b**–**12b**. Data are shown as mean ± SEM. Statistical significance was calculated using ANOVA analysis followed by Dunnett’s post hoc test (**** *p* < 0.0001 when compared to the negative control).

Genotoxicity screening is often overlooked in the initial phase of drug research and development process. Nevertheless, for toxoplasmosis chemotherapy, which can take 6 months or longer in immunodeficient patients or even 12 months in congenitally infected newborns, it is crucial to rule out genotoxic compounds at early stage of drug development, before more costly and time-effective assays are involved. Therefore, for our best anti-*Toxoplasma* candidates, **10b**–**12b**, their possible genotoxic activities were investigated in the single cell gel electrophoresis (SCGE) assay. This assay, also known as comet assay, enables one to detect DNA damage in individual eukaryotic cells. Briefly, when a compound is genotoxic, its intercalation or interaction with the genomic DNA can lead to DNA strand breaks and fragmentation. After lysis of the cells, the possible DNA fragmentation is measured by submitting the cell nuclei to electrophoresis. Through pore exclusion of agarose, small DNA fragments will migrate further (comet tail) than larger fragments or undamaged DNA strands (comet head). The possible genotoxic activities of **10b**–**12b** were measured after 24 h exposition of Hs27 cells to the respective CC_30_ concentrations. The comet assay was performed under alkaline conditions, in which different types of DNA damage can be detected (e.g., DNA single- and double-strand breaks, DNA-DNA cross-linking, etc.). Degradation of DNA was assessed both qualitatively (Figure 5) and quantitatively (Figure 6), as %DNA in the comet tail. Cisplatin (10 µg/mL) was used as a positive control. As can be seen in Figure 6, none of the tested compounds exhibited genotoxic activity. There were no statistically significant differences between %DNA in comet tail of untreated cells when compared to Hs27 cells exposed to **10b**–**12b**.

### 3.4. Influence of the Thiadiazoles during Toxoplasma Growth

**Figure 7:**
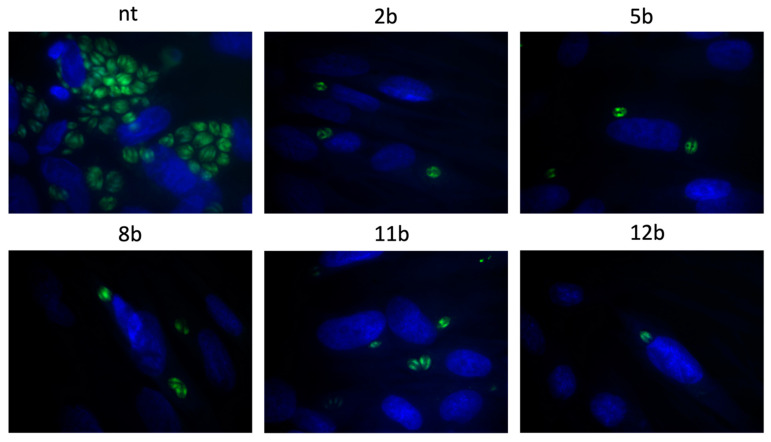
Representative images from Assay A. Host cells infected with tachyzoites for 3 h in the milieu of the 1,3,4-thiadiazoles **2b**, **5b**, **8b**, **11b** or **12b** and then washed, incubated for 24 h in free medium; *T. gondii*—green, cell nuclei—blue; NT—non-treated.

**Figure 8:**
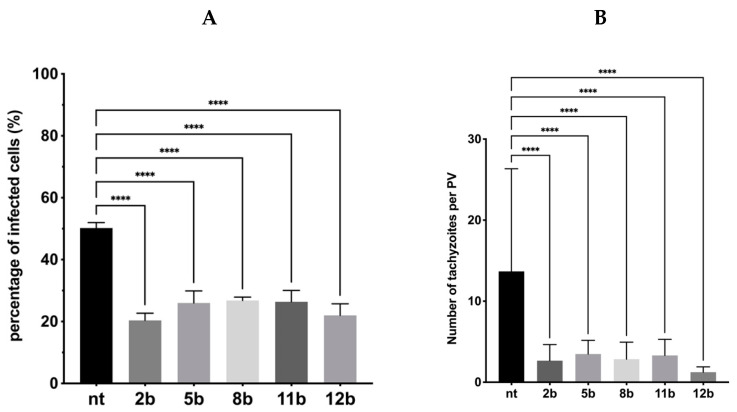
Influence of the 1,3,4-thiadiazoles **2b**, **5b**, **8b**, **11b** and **12b** during *Toxoplasma* tachyzoites growth (Assay A). Host cells infected with tachyzoites for 3 h in the milieu of **2b**, **5b**, **8b**, **11b** or **12b** and then washed, incubated for 24 h in free medium. (**A**) Percentage of infected cells. (**B**) Number of tachyzoites per PV. PV—parasitophorous vacuole; nt—non-treated. Data shown are the combined averages of three independent experiments on triplicate chamber slides under the same conditions. Error bars indicate standard error of the mean. Values with statistically significant differences are labeled by brackets and asterisks as follows: **** *p* < 0.0001. Data were compared using a two-way ANOVA followed by Dunnett’s multiple comparisons test.

As mentioned in the Introduction, although initial interest has been oriented towards 1,3,4-thiadiazoles as potential anti-*Toxoplasma* agents [26], to date nothing is known about molecular mechanism underlying their anti-*Toxoplasma* activity. To address this missing knowledge, we selected the most potent compounds **2b**, **5b**, **8b**, **11b** and **12b** to test their efficacy in *Toxoplasma* tachyzoites invasion and subsequent intracellular proliferation assays. *T. gondii* establishes its lytic growth cycle in host cells through the active process of invasion. This process, essential for the pathogenicity of *T. gondii*, is initiated by attachment of the tachyzoites to the cell and ordinarily quickly progresses to penetration of that cell [31]. Thus, in the first variant of the experiments (Assay A), the effect of **2b**, **5b**, **8b**, **11b** and **12b** toward penetration of tachyzoites into the host cells was determined. In this assay, host cells were infected for 3 h by tachyzoites in the milieu of tested compound and then washed out. Next, the cultures were incubated for following 24 h in normal medium. Thus, this assay differentiates between compounds that can act extracellularly, i.e., directly on the parasite, from those that require being in the intracellular environment, i.e., inside the host cell, to manifest anti-*Toxoplasma* effect [32]. As presented in Figure 7, the 1,3,4-thiadiazoles **2b**, **5b**, **8b**, **11b** and **12b** appear to have the ability to operate extracellularly. Specifically, data from microscopic analysis followed by Dunnett’s multiple comparisons test after two-way ANOVA (Figure 8) found that the compounds caused significant reductions in both percentage of infected cells and in the number of tachyzoites per vacuole compared to non-treated host cells.

**Figure 9:**
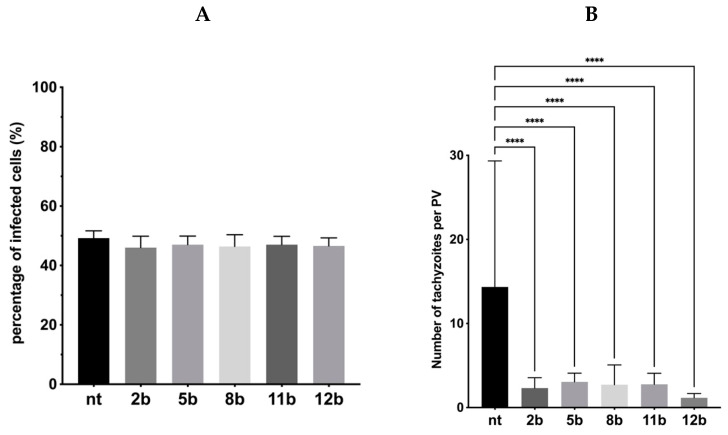
Influence of the 1,3,4-thiadiazoles **2b**, **5b**, **8b**, **11b** and **12b** during *Toxoplasma* tachyzoites growth (Assay B). Host cells infected with tachyzoites for 3 h in free medium, then washed and incubated 24 h in the milieu of **2b**, **5b**, **8b**, **11b** or **12b**. (**A**) Percentage of infected cells. (**B**) Number of tachyzoites per PV. PV—parasitophorous vacuole, nt—non-treated. Data shown are the combined averages of three independent experiments on triplicate chamber slides under the same conditions. Error bars indicate standard error of the mean. Values with statistically significant differences are labeled by brackets and asterisks as follows: ****, *p* < 0.0001. Data were compared using a two-way ANOVA followed by Dunnett’s multiple comparisons test.

**Figure 10:**
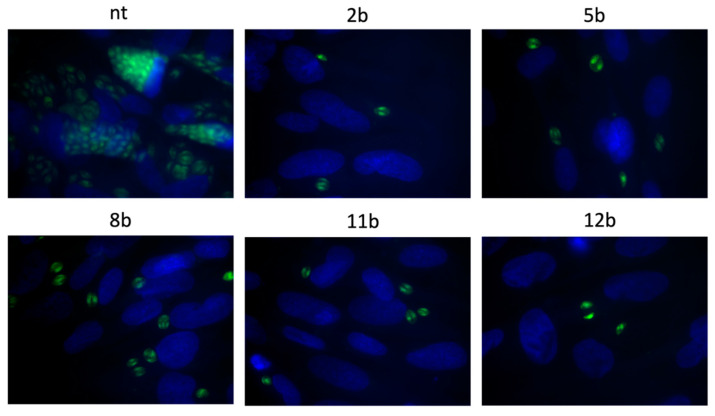
Representative images from assay B. Impeding tachyzoites division in host cells after 24 h incubation with tested 1,3,4-thiadiazoles **2b**, **5b**, **8b**, **11b** or **12b**; *T. gondii*—green, cell nuclei—blue; NT—not-treated.

Subsequently, the 1,3,4-thiadiazoles **2b, 5b, 8b, 11b and 12b** were submitted to assay B followed by Dunnett’s multiple comparisons test after two-way ANOVA to determine the compound’s ability to inhibit an intracellular proliferation. In this assay, tachyzoites were cultured with host cells for 3 h, in the absence of tested compounds in the milieu, and then washed out. Next, they were incubated for following 24 h in the presence of **2b**, **5b**, **8b**, **11b** and **12b**. Thus, this assay points the compounds that can act intracellularly, i.e., those that require being in the intracellular environment, inside the host cell, to manifest antiparasitic activity. As we expected, the percentage of infected cells was still the same (Figure 9) due to the fact tachyzoites had 3 h to enter the cell and form parasitophorous vacuole. However, the significant reduction in both size of parasitophorous vacuole and number of intravacuolar tachyzoites was observed (Figure 10). Thus, the compounds **2b**, **5b**, **8b**, **11b** and **12b** significantly inhibit the parasite proliferation by impeding tachyzoites division.

### 3.5. Effects of the Thiadiazoles on the Kinetics of Toxoplasma gondii Growth

**Figure 11:**
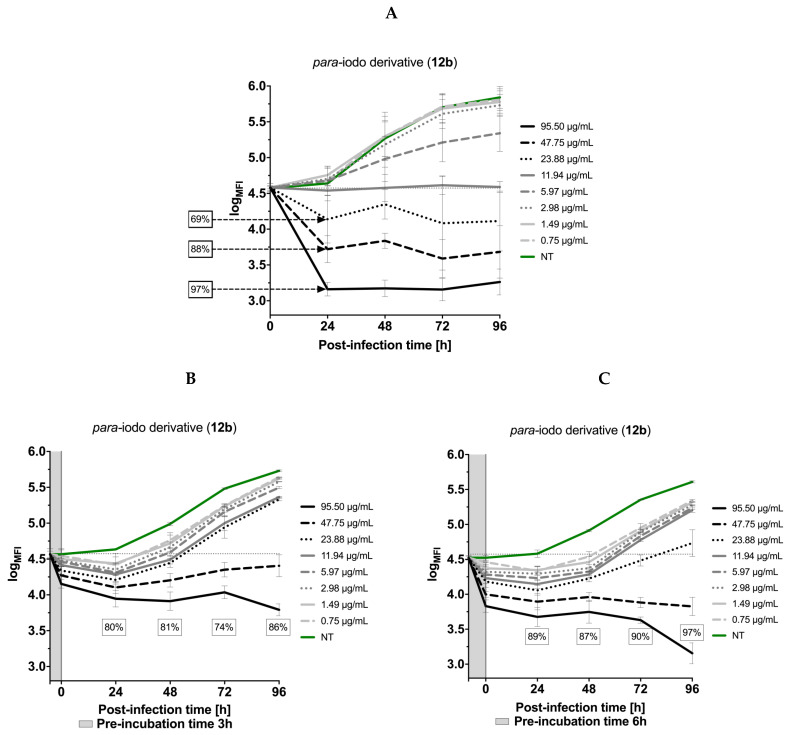
The kinetics of *T. gondii* growth. Concentration and time dependent curves of 1,3,4-thiadiazoles derivative **12b** against *T. gondii* RH-GFP strain. (**A**) Tachyzoites were given 1 h to allow them to infect host cells in the absence of compound and then were exposed to **12b** for 96 h at 37 °C. Fluorescence measurement was performed every 24 h. (**B**,**C**) Tachyzoites were exposed to **12b** for 3 and 6 h (pre-incubation time) at 37 °C, then added to the Hs27 cells, incubated for 96 h at 37 °C. Fluorescence measurement was performed every 24 h. Experiments were performed in triplicate, data presented as logarithm of mean fluorescence intensity (log_MFI_). MFI—mean fluorescence intensity.

Finally, the effects of the most potent compound **12b** (SI = 20.89) on the kinetics of *T. gondii* growth was tested. It was observed that **12b** showed concentration-dependent antiparasitic activity (Figure 11A–C). As presented in Figure 11A (Assay 1), the compound at the concentration of 250 µM (95.50 µg/mL) inhibited the growth of intracellular tachyzoites by 97% during the first 24 h post-infection and this level of inhibition was maintained for the next 72 h. Additionally, the influence of **12b** on extracellular tachyzoites was tested (Assay 2; Figure 11B,C). It was found that the extension of the preincubation time of tachyzoites (by 3 or 6 h) with 12b dramatically reduced the number of the newly formed parasites in Hs27 host cells already 24 h after infection, to 80 and 89%, respectively. Moreover, extended time of incubation up to 96 h resulted in an even stronger impact on *T. gondii*, which finally decreased proliferation to 89% and 97% (Figure 11B,C). Summarizing, these data are very promising, because the observed inhibition of *T. gondii* proliferation in the presence of **12b** indicated that putative target protein for **12b** or one of the metabolic pathways could be probably inhibited. The confirmation of an in vivo efficacy and the determination of the optimal dosage will be a task for upcoming studies in the animal model.

**Figure 12:**
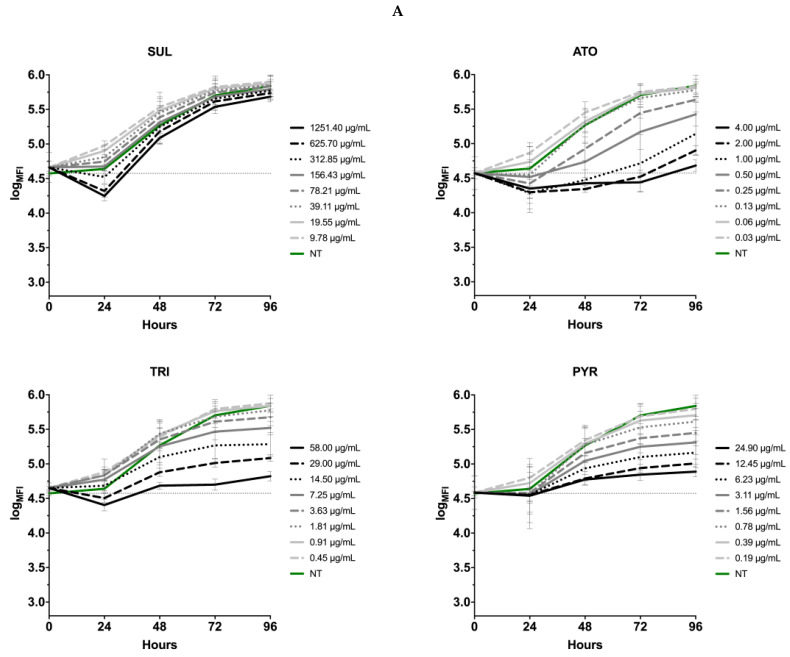

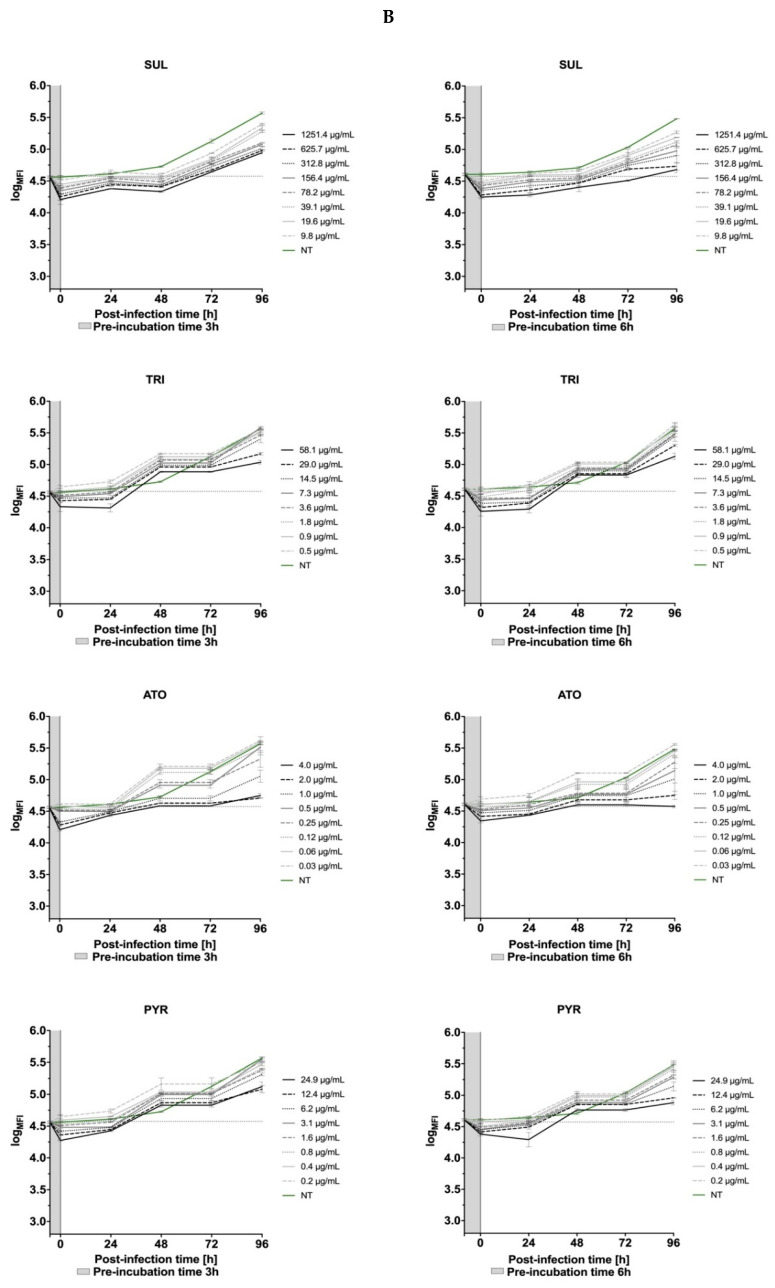
The kinetics of *T. gondii* growth. Concentration and time dependent curves of drugs against *T. gondii* RH- GFP strain. (**A**) Tachyzoites were given 1 h to allow them to infect host cells in the absence of compound and then were exposed to compounds for 96 h at 37 °C. Fluorescence measurement was performed every 24 h. (**B**) Tachyzoites were exposed to drugs for 3 and 6 h at 37 °C, then added to the Hs27 cells, incubated for 96 h at 37 °C. Fluorescence measurement was performed every 24 h. Experiments were performed in triplicate, data presented as logarithm of mean fluorescence intensity (log_MFI_). MFI—mean fluorescence intensity; SUL—sulfadiazine; TRI—trimethoprim; ATO—atovaquone; PYR—pyrimethamine.

The effect of drugs (SUL, TRI, PYR and ATO) on the kinetics of *T. gondii* growth was also tested. It was found that the inhibitors of folic acid synthesis and atovaquone only moderately slow down the proliferation of tachyzoites. None of the commercial drugs used in our study produced such significant effects in the level of *T. gondii* inhibition as **12b** (Figure 12).

## 4. Conclusions

We synthesized a series of 1,3,4-thiadiazole-2-halophenylamines functionalized at the C5 position with the imidazole ring to assess their inhibitory activity against *Toxoplasma gondii* parasite. All of them stood out as promising antiparasitic agents possessing an activity similar or better than that of sulfadiazine and trimethoprim, and better selectivity index with respect to these reference drugs. Furthermore, we also documented that the most active compounds effectively blocked the parasite invasion and intracellular proliferation via direct action on both tachyzoites and parasitophorous vacuoles formation. Finally, it is of note that the most promising compounds with iodo substitution are non-genotoxic. Collectively, these results demonstrated the importance of the 1,3,4-thiadiazole scaffold for the development of new anti-*Toxoplasma* drugs. However, in vivo studies are required for a complete evaluation of their efficacy. Such experiments are currently underway in our research laboratory.

## Data Availability

Data are contained within the article or supplementary materials.

## Supplementary Materials

The following are available online at https://www.mdpi.com/article/10.3390/cells10051053/s1, Synthesis of the 1,3,4-thiadiazoles **1b**–**12b**, Supplementary Figure S1: Dose response curves and IC_50_ values (μg/mL) for the 1,3,4-thiadiazoles **1b**–**12b** on *T. gondii* proliferation, Supplementary Table S1: The cytotoxic concentration (CC_30_, [µg/mL]), inhibitory concentration (IC_50_, (µg/mL)), and the selectivity index (SI) of the thiosemicarbazides **1a**–**12a** and the 1,3,4-thiadiazoles **1b**–**12b**, Supplementary Table S2: Log*P* and logD values for the thiosemicarbazides **1a**–**12a** and the 1,3,4-thiadiazoles **1b**–**12b**.
