# Supplementary material for: 1,3,4-Thiadiazoles Effectively Inhibit Proliferation of Toxoplasma gondii
Source: Cells. 2021 Apr 29;10(5):1053. doi: 10.3390/cells10051053 (PMC8145641; doi:10.3390/cells10051053)
Supplement: Supplementary file 1 [file cells-10-01053-s001.zip › Supplementary Materials.pdf]

cells

MDPI

Supplementary Materials

1,3,4-Thiadiazoles effectively block invasion and inhibit proliferation of *Toxoplasma gondii*

Lidia Węglińska [1], Adrian Bekier [2], Katarzyna Dzitko [2,*], Barbara Pacholczyk-Sienicka [3], Łukasz Albrecht [3], Tomasz Plech [4], Piotr Paneth [5,6] and Agata Paneth [1,*]

[1] Department of Organic Chemistry, Faculty of Pharmacy, Medical University of Lublin, Chod´zki 4a, 20-093 Lublin, Poland; lidia.weglinska@umlub.pl
[2] Department of Molecular Microbiology, Faculty of Biology and Environmental Protection, University of Lodz, Banacha 12/16, 90-237 Lodz, Poland; adrian.bekier@biol.uni.lodz.pl
[3] Institute of Organic Chemistry, Faculty of Chemistry, Lodz University of Technology, Zeromskiego 116, 90-924 Lodz, Poland; barbara.pacholczyk@p.lodz.pl (B.P.-S.); lukasz.albrecht@p.lodz.pl (Ł.A.)
[4] Department of Pharmacology, Faculty of Health Sciences, Medical University of Lublin, Chodzki 4a, 20-093 Lublin, Poland; tomasz.plech@umlub.pl
[5] Institute of Applied Radiation Chemistry, Lodz University of Technology, Zeromskiego 116, 90-924 Lodz, Poland; piotr.paneth@p.lodz.pl
[6] International Centre for Research on Innovative Biobased Materials (ICRI-BioM)—International Research Agenda, Lodz University of Technology, Zeromskiego 116, 90-924 Lodz, Poland
* Correspondence: katarzyna.dzitko@biol.uni.lodz.pl (K.D.); agata.paneth@umlub.pl (A.P.)

page 2: Synthesis of the 1,3,4-thiadiazoles **1b–12b**

page 5: **Figure S1.** Dose response curves and IC_{50} values [μg/mL] for the 1,3,4-thiadiazoles **1b–12b** on *T. gondii* proliferation.

page 6: **Table S1.** The cytotoxic concentration (CC_{30}, [μg/mL]), inhibitory concentration (IC_{50}, [μg/mL]), and the selectivity index (SI) of the thiosemicarbazides **1a–12a** and the 1,3,4-thiadiazoles **1b–12b**.

Table S2. Log*P* and logD values for the thiosemicarbazides **1a–12a** and the 1,3,4-thiadiazoles **1b–12b**.

Synthesis of the 1,3,4-thiadiazoles 1b–12b

General. The prepared compounds were characterized by ^{1}H, ^{13}C NMR, and IR. Each of the tested compounds had ≥96% purity, as determined using elemental analysis. All chemicals used in the syntheses were obtained from Sigma-Aldrich (Saint Louis, MS, USA) and Alfa Aesar (Karlsruhe, Germany) and were used as received. TLC separations were performed on Merck aluminium plates with silica gel 60 F_{254}(Darmstadt, Germany). Melting points were determined on a Fischer-Johns block and are uncorrected. Elemental analyses were determined by a AMZ-CHX elemental analyzer (PG, Gdańsk, Poland). All ^{1}H NMR spectra were recorded using a Bruker Avance II Plus 16.4 T spectrometer (Bruker BioSpin, Germany) operating at the ^{1}H frequency 700.16MHz. The instrument was equipped with a 5 mm broadband BBI probe. An exponential line broadening of 0.05 Hz was applied to raw data prior to Fourier transformation. All samples were run at 300 K. Chemical shifts are reported as δ values in parts per million (ppm) and were indirectly referenced to the DMSO-d_6 signal at 2.50 ppm. All spectral regions were individually corrected using a fifth–order baseline function. Experimental studies were conducted in extra dry DMSO-$d_6$due to the fact that the signals of the NH protons were otherwise not observed because of rapid proton exchange and overlapping with the water signal. Extra dry solvent was prepared from a commercial sample by drying over freshly activated 4Å molecular sieves. All 2D heteronuclear experiments were carried out with the pulse field gradient. IR spectra were recorded by Nicolet 6700 spectrometer (Thermo Scientific, Philadephia, PA, USA).

Synthetic protocol for the 1,3,4-thiadiazoles 1b–12b. A mixture of corresponding thiosemicarbazide **1a–12a** (0.01 mol) and 3 mL of concentrated H_2SO_4 was left for 2 h at room temperature. The resulting solution was next poured into crushed ice. The precipitate was collected by filtration, washed with water, and crystallized from buthanol.

N-(2-fluorophenyl)-5-(4-methylimidazole-5-yl)-1,3,4-thiadiazole-2-amine (**1b**). Yield: 33% (white solid); mp 257–258 °C. ^{1}H NMR (700MHz, DMSO-*d*$_6$): *δ* 10.43 (br s, 1H, NH-imidazole), 8.91 (s, 1H, *H*-imidazole), 8.37 (td, 1H, *J* = 1.5 and 8.4 Hz, H-Ar), 7.30 (ddd, 1H, *J* = 1.5, 8.4, and 11.5 Hz, H-Ar), 7.23 (td, 1H, *J* = 1.2 and 7.9 Hz, H-Ar), 7.09 (m, 1H, H-Ar), 4.69 (br s, 1H, NHPh), 2.53 (s, 3H, CH_3). ^{13}C NMR (176.1 MHz, DMSO-*d*$_6$): *δ* 167.93 (C_{IV} thiadiazole), 152.57 (d, *J* = 244.3 Hz, C_{IV} Ar), 148.81 (C_{IV} thiadiazole), 135.30 (C_2 imidazole), 128.95 (C_4 imidazole), 128.66 ($C_{IV,}$ *J* = 11.3 Hz, Ar), 125.29 (d, *J* = 3.5Hz, CH Ar), 123.99 (d, *J* = 7.1 Hz, CH Ar), 121.73 (C_5 imidazole), 121.21 (CH Ar), 115.84 (d, *J* = 18.9 Hz, CH Ar), 10.88 (CH_3). IR (ν_{max} cm^{-1}) 3454.22, 3015.99, 2970.06, 1738.26, 1620.98, 1597.20, 1556.43, 1507.92, 1483.34, 1457.59, 1365.47, 1256.36, 1228.40, 1216.85, 1204.20, 1048.69, 861.34, 759.81, 575.99.Anal. Calcd for $C_{12}H_{10}FN_5S$:C 52.35; H 3.66; N 25.44. Found: C 52.28; H 3.55; N 25.32.

N-(3-fluorophenyl)-5-(4-methylimidazole-5-yl)-1,3,4-thiadiazole-2-amine (**2b**). Yield: 42% (white solid); mp 268–269 °C. ^{1}H NMR (700 MHz, DMSO-*d*$_6$) *δ* 10.72 (br s, 1H, NH-imidazole), 8.40 (s, 1H, *H*-imidazole), 7.72 (dt, 1H, *J* = 2.2 and 11.8 Hz, H-Ar), 7.38 (q, 1H, *J* = 8.2 Hz, H-Ar), 7.32 (dd, 1H, *J* = 2.2 and 8.2 Hz, H-Ar), 6.82 (td, 1H, *J* = 2.2 and 8.2 Hz, H-Ar), 5.09 (br s, 1H, NHPh), 2.53 (s, 3H, CH_3). ^{13}C NMR (176.1 MHz, DMSO-*d*$_6$): *δ* 163.52 (C_{IV} thiadiazole), 163.06 (d, *J* = 241.0 Hz, C_{IV} Ar), 151.78 (C_{IV} thiadiazole), 142.61 ($C_{IV,}$ *J* = 11.4Hz, Ar), 135.38 (C_2 imidazole), 131.15 (d, *J* = 9.9 Hz, CH Ar), 128.12 (C_4 imidazole), 124.5 (C_5 imidazole), 113.81 (CH Ar), 108.73 (d, *J* = 21.1 Hz, CH Ar), 104.82 (d, *J* = 26.8 Hz, CH Ar), 10.97 (CH_3). IR (ν_{max} cm^{-1}) 3158.51, 2998.32, 2970.02, 1738.47, 1598.37, 1557.98, 1491.88, 1447.37, 1229.13, 1217.00, 1205.78, 1137.29, 1065.41, 958.10, 895.61, 866.46, 840.82, 774.20, 763.39. Anal. Calcd for $C_{12}H_{10}FN_5S$: C 52.35; H 3.66; N 25.44. Found: C 51.98; H 3.56; N 25.46.

N-(4-fluorophenyl)-5-(4-methylimidazole-5-yl)-1,3,4-thiadiazole-2-amine (**3b**). Yield: 42% (white solid); mp 165-166 °C (Lit. [26] 259–261 °C). ^{1}H NMR (700MHz, DMSO-*d*$_6$):*δ* 10.68 (br s, 1H, NH-imidazole), 9.13 (s, 1H, *H*-imidazole), 7.69 (dd, 2H, *J* = 4.8 and 8.9 Hz, H-Ar), 7.21 (t, 2H, *J* = 8.9 Hz, H-Ar), 6.78 (br s, 1H, NHPh), 2.51 (s, 3H, CH_3). ^{13}C NMR

(176.1 MHz, DMSO-d_6): δ 165.28 (C_{IV} thiadiazole), 158.04 (d, J = 239.3 Hz, C_{IV} Ar), 145.91 (C_{IV} thiadiazole), 137.19 ($C_{IV,}$ J = 2.5Hz, Ar), 135.24 (C_2 imidazole), 129.23 (C_4 imidazole), 120.61 (C_5 imidazole), 119.99 (d, J = 7.5Hz, 2×CH Ar), 116.19 (d, J = 21.7 Hz, 2×CH Ar), 10.52 (CH_3). IR (ν_{max} cm^{-1}) 3445.79, 3117.03, 3027.67, 2969.93, 1738.44, 1628.08, 1596.88, 1503.13, 1365.40, 1228.21, 1216.83, 1154.28, 1034.83, 837.81, 575.81. Anal. Calcd for $C_{12}H_{10}FN_5S$: C 52.35; H 3.66; N 25.44. Found: C 52.55; H 3.59; N 25.74.

N-(2-chlorophenyl)-5-(4-methylimidazole-5-yl)-1,3,4-thiadiazole-2-amine (**4b**). Yield: 26% (white solid); mp 235–236 °C. ^{1}H NMR (700 MHz, DMSO-d_6): δ 11.91 (br s, 1H, NH-imidazole), 9.05 (s, 1H, *H*-imidazole), 8.28 (dd, 1H, J = 1.4, 7.9 Hz, H-Ar), 7.54 (dd, 1H, J = 1.4, 7.9 Hz, H-Ar), 7.41 (td, 1H, J = 1.4, 7.9 Hz, H-Ar), 7.15 (td, 1H, J = 1.4, 7.9 Hz, H-Ar), 6.81 (br s, 1H, NHPh), 2.52 (s, 3H, CH_3). ^{13}C NMR (176.1 MHz, DMSO-d_6): δ 165.88 (C_{IV} thiadiazole), 147.68 (C_{IV} thiadiazole), 137.41 (C_{IV} Ar), 135.28 (C_2 imidazole), 130.30 (CH Ar), 129.23 (C_4 imidazole), 128.47 (CH Ar), 125.35 (CH Ar), 124.00 (C_{IV}, Ar), 122.73 (CH Ar), 120.91 (C_5 imidazole), 10.83 (CH_3). IR (ν_{max} cm^{-1}) 3251.69, 3016.09, 2970.03, 1738.36, 1597.96, 1576.54, 1544.55, 1495.43, 1446.87, 1365.33, 1228.39, 1216.02, 1150.79, 1032.36, 868.65, 752.32, 571.81. Anal. Calcd for $C_{12}H_{10}ClN_5S$:C 49.40; H 3.45; N 24.00. Found: C 49.23; H 3.51; N 23.92.

N-(3-chlorophenyl)-5-(4-methylimidazole-5-yl)-1,3,4-thiadiazole-2-amine (**5b**). Yield: 47% (white solid); mp 185–186 °C. ^{1}H NMR (700 MHz, DMSO-d_6): δ 10.70 (br s, 1H, NH-imidazole), 8.43 (s, 1H, *H*-imidazole), 7.95 (t, 1H, J = 2.1, H-Ar), 7.47 (ddd, 1H, J = 0.8, 2.1, 8.0 Hz, H-Ar), 7.38 (t, 1H, J = 8.1, H-Ar), 7.06 (ddd, 1H, J = 0.8, 2.1, 8.0 Hz, H-Ar), 3.99 (br s, 1H, NHPh), 2.53 (s, 3H, CH_3). ^{13}C NMR (176.1 MHz, DMSO-d_6): δ 163.52 (C_{IV} thiadiazole), 151.59 (C_{IV} thiadiazole), 142.27 (C_{IV} Ar), 135.38 (C_2 imidazole), 133.98 (C_{IV}, Ar), 131.16 (CH Ar), 128.21 (C_4 imidazole), 124.09 (C_5 imidazole), 121.98 (CH Ar), 117.32 (CH Ar), 116.40 (CH Ar), 10.96 (CH_3). IR (ν_{max} cm^{-1}) 3263.14, 3015.83, 1738.17, 1597.86, 1497.65, 1430.07, 1085.56, 690.72, 779.51, 627.50. Anal. Calcd for $C_{12}H_{10}ClN_5S$: C 49.40; H 3.45; N 24.00. Found: C 49.58; H 3.51; N 24.05.

N-(4-chlorophenyl)-5-(4-methylimidazole-5-yl)-1,3,4-thiadiazole-2-amine (**6b**). Yield: 40% (white solid); mp 215-216 °C (Lit. [26] 256–257 °C). ^{1}H NMR (700 MHz, DMSO-d_6): δ 10.93 (br s, 1H, NH-imidazole), 8.53 (s, 1H, *H*-imidazole), 7.70 (d, 2H, J = 8.9, H-Ar), 7.41 (d, 2H, J = 8.9 Hz, H-Ar), 4.96 (br s, 1H, NHPh), 2.53 (s, 3H, CH_3).^{13}C NMR (176.1 MHz, DMSO-d_6): δ 163.89 (C_{IV} thiadiazole), 150.28 (C_{IV} thiadiazole), 139.85 (C_{IV} Ar), 135.33 (C_2 imidazole), 129.41 (2×CH Ar), 128.35 (C_4 imidazole), 125.96 (C_{IV}, Ar), 123.44 (C_5 imidazole), 119.52 (2×CH Ar), 10.93 (CH_3). IR (ν_{max} cm^{-1}) 3282.18, 3159.63, 1738.43, 1595.06, 1481.49, 1431.48, 1374.12, 1228.21, 1217.09, 1204.67, 1060.24, 956.57, 903.47, 841.31, 828.78, 799.86, 563.72. 499.63. Anal. Calcd for $C_{12}H_{10}ClN_5S$: C 49.40; H 3.45; N 24.00. Found: C 49.41; H 3.35; N 23.98.

N-(2-bromophenyl)-5-(4-methylimidazole-5-yl)-1,3,4-thiadiazole-2-amine (**7b**). Yield: 30% (white solid); mp 148–149 °C. ^{1}H NMR (700 MHz, DMSO-d_6): δ 11.80 (br s, 1H, NH-imidazole), 8.92 (s, 1H, *H*-imidazole), 8.11 (d, 1H, J = 7.9 Hz, H-Ar), 7.71 (d, 1H, J = 7.9 Hz, H-Ar), 7.44 (t, 1H, J = 7.9 Hz, H-Ar), 7.11 (t, 1H, J = 7.9 Hz, H-Ar), 5.50 (br s, 1H, NHPh), 2.51 (s, 3H, CH_3). ^{13}C NMR (176.1 MHz, DMSO-d_6): δ 166.14 (C_{IV} thiadiazole), 148.64 (C_{IV} thiadiazole), 139.01 (C_{IV} Ar), 135.28 (C_2 imidazole), 133.64 (CH Ar), 129.08 (CH Ar), 128.82 (C_4 imidazole), 126.32 (CH Ar), 124.00 (CH Ar), 121.82 (C_5 imidazole), 115.52 (C_{IV}, Ar), 10.86 (CH_3). IR (ν_{max} cm^{-1}) 2888.59, 1654.43, 1586.84, 1534.76, 1436.85, 1296.77, 1170.42, 1022.77, 927.81, 756.44, 575.17. Anal. Calcd for $C_{12}H_{10}BrN_5S$: C 42.87; H 3.00; N 20.83. Found: C 42.95; H 3.05; N 20.89.

N-(3-bromophenyl)-5-(4-methylimidazole-5-yl)-1,3,4-thiadiazole-2-amine (**8b**). Yield: 54% (white solid); mp 295–296 °C. ^{1}H NMR (700 MHz, DMSO-d_6): δ 10.81 (br s, 1H, NH-imidazole), 8.85 (s, 1H, *H*-imidazole), 8,08 (t, 1H, J = 2.0, H-Ar), 7.52 (ddd, 1H, J = 0.8, 2.0, 8.1 Hz, H-Ar), 7.33 (t, 1H, J = 8.1, H-Ar), 7.21 (ddd, 1H, J = 0.8, 2.0, 8.1 Hz, H-Ar), 5.24 (br s, 1H, NHPh), 2.54 (s, 3H, CH_3). ^{13}C NMR (176.1 MHz, DMSO-d_6): δ 164.12 (C_{IV} thiadiazole), 148.94 (C_{IV} thiadiazole), 142.22 (C_{IV} Ar), 135.36 (C_2 imidazole), 131.51 (CH Ar), 128.92 (C_4 imidazole), 125.14 (CH Ar), 122.47 (C_{IV} Ar), 122.13 (C_5 imidazole), 120.29 (CH

Ar), 116.91 (CH Ar), 10.91 (CH_3). IR (ν_{max} cm^{-1}) 3266.52, 3078.38, 1642.86, 1611.70, 1539.99, 1506.98, 1469.66, 1424.28, 1175.88, 1040.29, 860.98, 773.71, 573.17. Anal. Calcd for $C_{12}H_{10}BrN_5S$: C 42.87; H 3.00; N 20.83. Found: C 42.85; H 2.89; N 20.53.

N-(4-bromophenyl)-5-(4-methylimidazole-5-yl)-1,3,4-thiadiazole-2-amine (**9b**). Yield: 29% (white solid); mp 278–279 °C. ^{1}H NMR (700 MHz, DMSO-d_6): δ 10.62 (br s, 1H, NH-imidazole), 8.42 (s, 1H, *H*-imidazole), 7.64 (d, 2H, *J* = 8.5, H-Ar), 7.53 (d, 2H, *J* = 8.5 Hz, H-Ar), 4.90 (br s, 1H, NHPh), 2.52 (s, 3H, CH_3).^{13}C NMR (176.1 MHz, DMSO-d_6): δ 163.60 (C_{IV} thiadiazole), 151.39 (C_{IV} thiadiazole), 140.31 (C_{IV} Ar), 135.35 (C_2 imidazole), 132.28 (2×CH Ar), 128.10 (C_4 imidazole), 124.31 (C_5 imidazole), 119.87 (2×CH Ar), 113.72 (C_{IV} Ar), 10.96 (CH_3). IR (ν_{max} cm^{-1}) 3159.32, 2990.99, 1632.80, 1590,48, 1550.63, 1480.80, 1431.21, 1065.53, 904.36, 798.25, 613.17, 490.63. Anal. Calcd for $C_{12}H_{10}BrN_5S$: C 42.87; H 3.00; N 20.83. Found: C 42.93; H 2.95; N 20.77.

N-(2-iodophenyl)-5-(4-methylimidazole-5-yl)-1,3,4-thiadiazole-2-amine (**10b**). Yield: 18% (white solid); mp 185–186 °C. ^{1}H NMR (700 MHz, DMSO-d_6): δ 11.34 (br s, 1H, NH-imidazole), 8.32 (s, 1H, *H*-imidazole), 7.94 (dd, 1H, *J* = 1.4, 7.9 Hz, H-Ar), 7.77 (dd, 1H, *J* = 1.4, 7.9 Hz, H-Ar), 7.44 (td, 1H, *J* = 1.4, 7.9 Hz, H-Ar), 6.98 (td, 1H, *J* = 1.4, 7.9 Hz, H-Ar), 4.05 (br s, 1H, NHPh), 2.49 (s, 3H, CH_3). ^{13}C NMR (176.1 MHz, DMSO-d_6): δ 166.08 (C_{IV} thiadiazole), 151.80 (C_{IV} thiadiazole), 142.99 (C_{IV} Ar), 140.04 (CH Ar), 135.24 (C_2 imidazole), 129.77 (CH Ar), 127.63 (C_4 imidazole), 127.29 (CH Ar), 124.88 (CH Ar), 124.72 (C_5 imidazole), 94.52 (C_{IV} Ar), 10.94 (CH_3). IR (ν_{max} cm^{-1}) 3479.47, 3347.90, 3272.65, 3128.38, 1632.01, 1585.75, 1531.13, 1494.88, 1482.93, 1433.21, 1295.95, 1160.45, 1088.64, 1034.07, 1012.85, 898.47, 737.71, 607.27, 445.86. Anal. Calcd for $C_{12}H_{10}IN_5S$: C 37.61, H 2.63, N 18.28. Found: C 37.56, H 2.68, N 18.08.

N-(3-iodophenyl)-5-(4-methylimidazole-5-yl)-1,3,4-thiadiazole-2-amine (**11b**). Yield: 48% (white solid); mp 245-246 °C. ^{1}H NMR (700 MHz, DMSO-d_6): δ 10.67 (br s, 1H, NH-imidazole), 8.68 (s, 1H, *H*-imidazole), 8,23 (t, 1H, *J* = 2.0, H-Ar), 7.55 (ddd, 1H, *J* = 0.8, 2.0, 8.1 Hz, H-Ar), 7.38 (ddd, 1H, *J* = 0.8, 2.0, 8.1 Hz, H-Ar), 7.16 (t, 1H, *J* = 8.1, H-Ar), 5.14 (br s, 1H, NHPh), 2.53 (s, 3H, CH_3). ^{13}C NMR (176.1 MHz, DMSO-d_6): δ 163.87 (C_{IV} thiadiazole), 149.92 (C_{IV} thiadiazole), 142.12 (C_{IV} Ar), 135.35 (C_2 imidazole), 131.52 (CH Ar), 131.03 (CH Ar), 128.60 (C_4 imidazole), 126.07 (CH Ar), 122.94 (C_5 imidazole), 117.28 (CH Ar), 95.46 (C_{IV} Ar), 10.94 (CH_3). IR (ν_{max} cm^{-1}) 3172.89, 1588.98, 1507.55, 1421.71, 1279.62, 1082.48, 992.02, 779.68, 573.28. Anal. Calcd for $C_{12}H_{10}IN_5S$: C 37.61, H 2.63, N 18.28. Found: C 37.55, H 2.66, N 18.38.

N-(4-iodophenyl)-5-(4-methylimidazole-5-yl)-1,3,4-thiadiazole-2-amine (**12b**). Yield: 49% (white solid); mp 183–184°C. ^{1}H NMR (700 MHz, DMSO-d_6): δ 10.72 (br s, 1H, NH-imidazole), 8.84 (s, 1H, *H*-imidazole), 7.69 (d, 2H, *J* = 8.8, H-Ar), 7.52 (d, 2H, *J* = 8.8 Hz, H-Ar), 5.39 (br s, 1H, NHPh), 2.53 (s, 3H, CH_3). ^{13}C NMR (176.1 MHz, DMSO-d_6): δ 164.22 (C_{IV} thiadiazole), 148.56 (C_{IV} thiadiazole), 140.58 (C_{IV} Ar), 138.15 (2×CH Ar), 135.32 (C_2 imidazole), 128.82 (C_4 imidazole), 122.15 (C_5 imidazole), 120.34 (2×CH Ar), 85.53 (C_{IV} Ar), 10.89 (CH_3). IR (ν_{max} cm^{-1}) 3185.30, 1605.12, 1566.63, 1489.64, 1181.71, 1007.18, 817.16, 672.23, 575.57, 498.06. Anal. Calcd for $C_{12}H_{10}IN_5S$: C 37.61, H 2.63, N 18.28. Found: C 37.51, H 2.68, N 17.93.

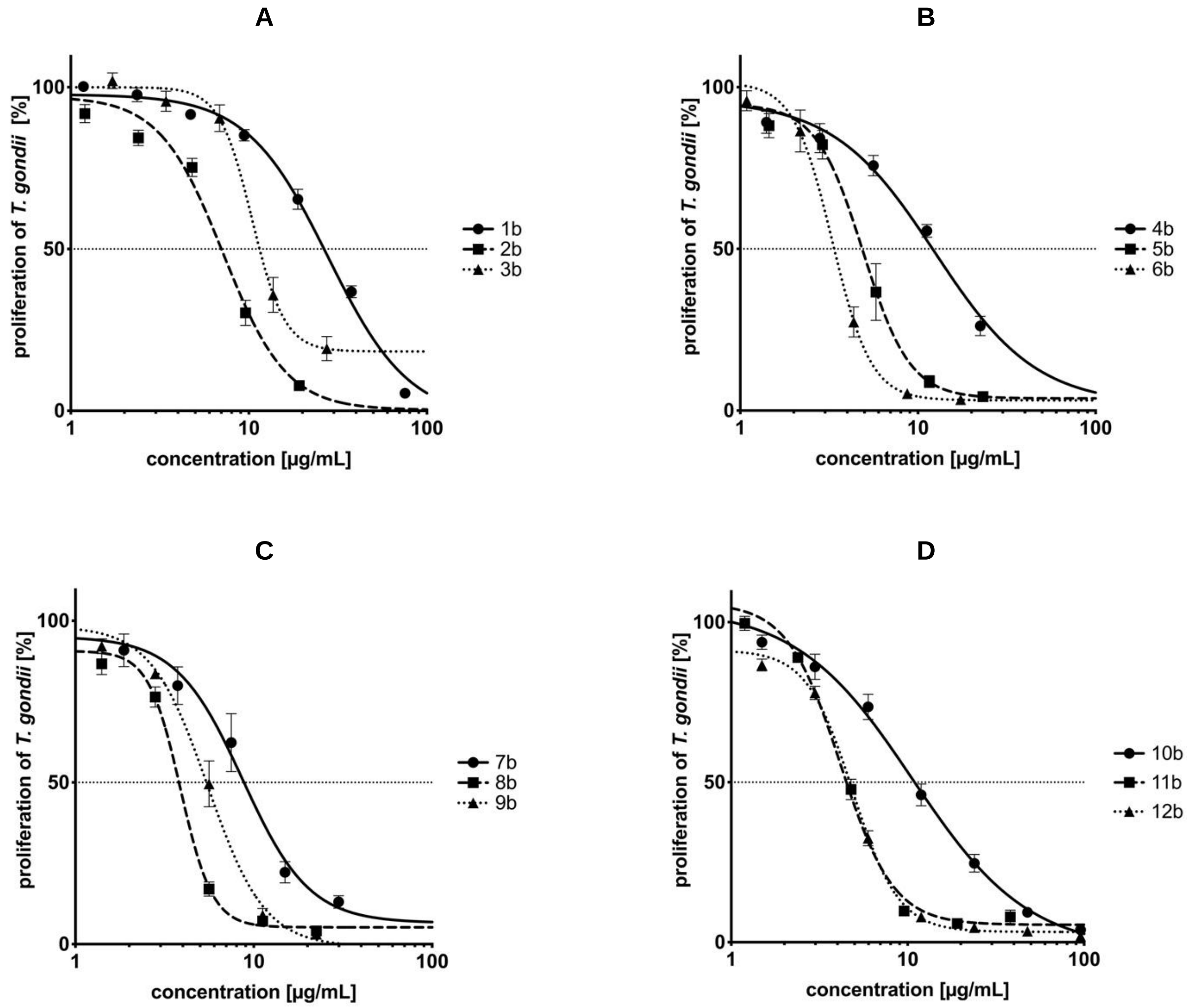

Figure S1. Dose response curves and IC_{50} values [µg/mL] for the 1,3,4-thiadiazoles **1b**–**12b** on *T. gondii* proliferation.

Table S1. The cytotoxic concentration (CC_{30}, [µg/mL]), inhibitory concentration (IC_{50}, [µg/mL]), and the selectivity index (SI) of the thiosemicarbazides **1a–12a** and the 1,3,4-thiadiazoles **1b–12b.**

thiosemicarbazide	CC_{30}	IC_{50}	SI	1,3,4-thiadiazole	CC_{30}	IC_{50}	SI
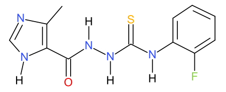 **1a**	289.60	68.83	6.61	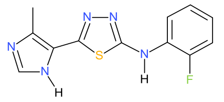 **1b**	157.71	26.17	6.03
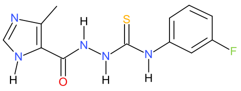 **2a**	125.17	113.45	7.79	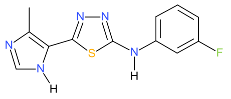 **2b**	18.96	7.04	2.69
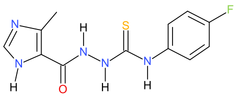 **3a**	266.20	110.31	8.86	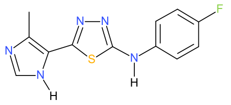 **3b**	26.17	11.43	2.29
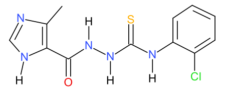 **4a**	143.15	35.61	17.69	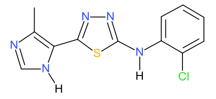 **4b**	24.69	12.37	2.00
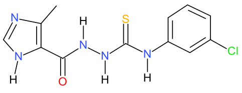 **5a**	96.78	24.15	11.30	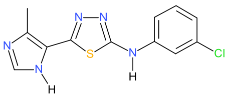 **5b**	17.88	3.65	4.90
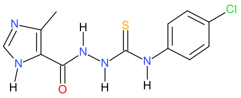 **6a**	94.12	73.37	3.15	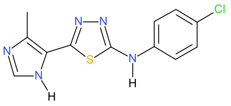 **6b**	45.97	8.36	5.50
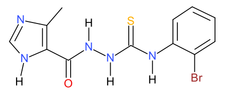 **7a**	205.31	27.65	17.91	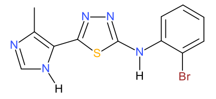 **7b**	29.71	8.77	3.39
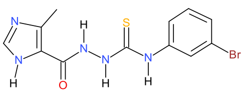 **8a**	96.12	15.64	15.20	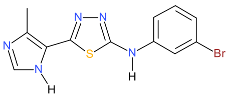 **8b**	20.55	3.83	5.37
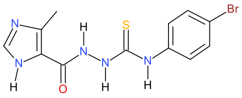 **9a**	48.89	14.57	15.35	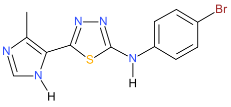 **9b**	21.52	5.41	3.98

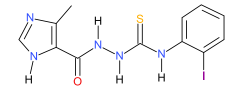 **10a**	101.58	20.31	9.79	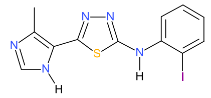 **10b**	89.54	11.02	8.13
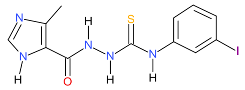 **11a**	85.57	10.30	19.16	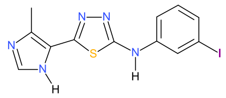 **11b**	36.48	4.49	8.13
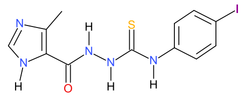 **12a**	71.58	22.01	8.86	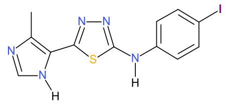 **12b**	98.18	4.70	20.89

positive controls:

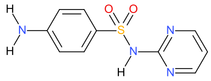 Sulfadiazine (SUL)	2230.61	1254.36	1.78	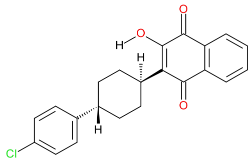 Atovaquone (ATO)	54.26	0.24	227.56
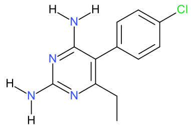 Pyrimethamine (PYR)	45.26	2.86	15.85	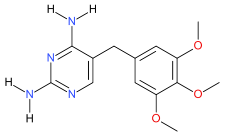 Trimethoprim (TRI)	17.78	14.79	1.20

CC_{30} [μg/mL] represents the non-cytotoxic concentration of the compound that caused only 30% of cells proliferation inhibition *in vitro*. The CC_{30} values were determined based on the plotted curves using GraphPad Prism program. IC_{50} [μg/mL] represents the concentration of the compound required for 50% inhibition of *T. gondii* proliferation *in vitro*. IC_{50} values were determined based on the plotted curves using GraphPad Prism program. The IC_{50} values for **1a**-**12a** were taken from ref. [25]. SI – selectivity index values were calculated as the ratio of the 30% cytotoxic concentration (CC_{30}) to the 50% antiparasitic concentration (IC_{50}).

Table S2. Log*P* and logD values for the thiosemicarbazides **1a–12a** and the 1,3,4-thiadiazoles **1b–12b.**

thiosemicarbazide	log*P*	logD	1,3,4-thiadiazole	log*P*	logD
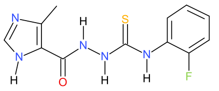 **1a**	1.54	1.10	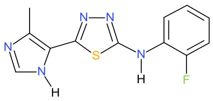 **1b**	2.68	1.97
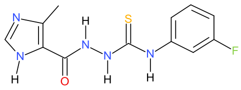 **2a**	1.61	1.10	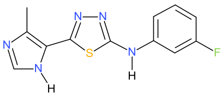 **2b**	2.64	1.97
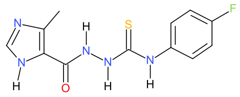 **3a**	1.52	1.10	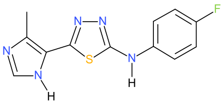 **3b**	2.65	1.97
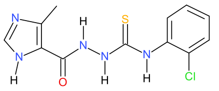 **4a**	1.75	1.56	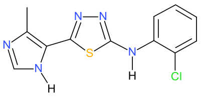 **4b**	2.90	2.43
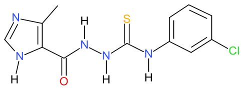 **5a**	1.92	1.56	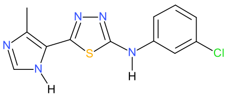 **5b**	2.87	2.43
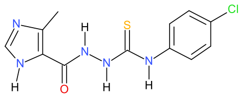 **6a**	1.80	1.56	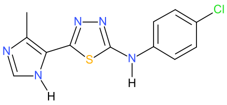 **6b**	2.87	2.43
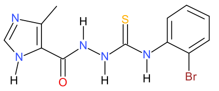 **7a**	1.85	1.73	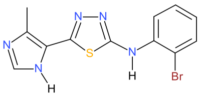 **7b**	2.99	2.59
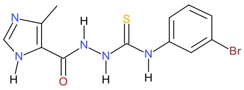 **8a**	1.90	1.73	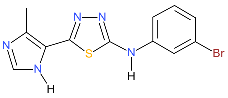 **8b**	2.95	2.59
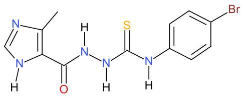 **9a**	1.90	1.73	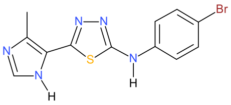 **9b**	3.01	2.59
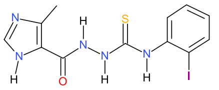 **10a**	1.89	1.89	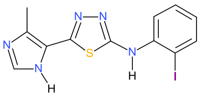 **10b**	3.02	2.75

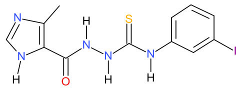 **11a**	1.96	1.89	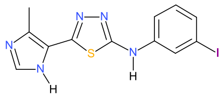 **11b**	3.00	2.75
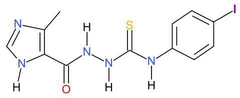 **12a**	1.91	1.89	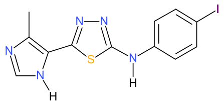 **12b**	3.00	2.75

Note: log*P* values were calculated using SwissADME program available online http://www.swissadme.ch/ logD values were calculated using logD Predictor available online https://disco.chemaxon.com/calculators/demo/plugins/logd/
